# Heart Rate Variability in Schizophrenia and Autism

**DOI:** 10.3389/fpsyt.2021.760396

**Published:** 2021-11-25

**Authors:** Sarah M. Haigh, Tabatha P. Walford, Pat Brosseau

**Affiliations:** ^1^Department of Psychology and Center for the Neural Basis of Cognition, Carnegie Mellon University, Pittsburgh, PA, United States; ^2^Department of Psychology and Center for Integrative Neuroscience, University of Nevada, Reno, Reno, NV, United States

**Keywords:** autism, schizophrenia, heart rate variability, electrocardiography, autonomic functioning

## Abstract

Suppressed heart rate variability (HRV) has been found in a number of psychiatric conditions, including schizophrenia and autism. HRV is a potential biomarker of altered autonomic functioning that can predict future physiological and cognitive health. Understanding the HRV profiles that are unique to each condition will assist in generating predictive models of health. In the current study, we directly compared 12 adults with schizophrenia, 25 adults with autism, and 27 neurotypical controls on their HRV profiles. HRV was measured using an electrocardiogram (ECG) channel as part of a larger electroencephalography (EEG) study. All participants also completed the UCLA Loneliness Questionnaire as a measure of social stress. We found that the adults with schizophrenia exhibited reduced variability in R-R peaks and lower low frequency power in the ECG trace compared to controls. The HRV in adults with autism was slightly suppressed compared to controls but not significantly so. Interestingly, the autism group reported feeling lonelier than the schizophrenia group, and HRV did not correlate with feelings of loneliness for any of the three groups. However, suppressed HRV was related to worse performance on neuropsychological tests of cognition in the schizophrenia group. Together, this suggests that autonomic functioning is more abnormal in schizophrenia than in autism and could be reflecting health factors that are unique to schizophrenia.

## Introduction

Schizophrenia and autism share many neuropsychological, symptom, and biological characteristics (1, 2), suggesting similar underlying physiological mechanisms may be driving or contributing to the conditions (3–5). However, understanding the perturbed mechanisms specific to each condition can reveal subtle markers of unique psychopathology. Altered autonomic functioning is common across both autism and schizophrenia and is related to several comorbid conditions including cardiac, gastrointestinal, and autoimmune disorders (6–8). A common measure of autonomic functioning that has been shown to be abnormal in both schizophrenia and autism is reduced heart rate variability (HRV) (9–11).

HRV refers to the variability in number of heartbeats within a given period. This variability is commonly interpreted to reflect autonomic functioning, with low variability reflecting a suppressed autonomic system (12, 13). HRV, therefore, provides a fast and simple measure of stress and cardiovascular health (14). HRV reduces with age (15, 16) and can indicate cognitive decline (17–20), while increased HRV has been linked to healthy aging and longevity (21). Therefore, HRV can reflect both neurological and physiological health. Both schizophrenia and autism exhibit reduced HRV (9–11). Isolating the unique HRV profile in each condition will help to identify improvements or decrements in health, and in what way both conditions deviate from the neurotypical trajectory.

In schizophrenia, reduced HRV is associated with increased symptom severity, worse scores on cognitive tests, and poorer quality of life (22, 23), and is independent of age and physical activity levels (24). Early childhood trauma and HRV predicted social functioning in schizophrenia (25), suggesting that HRV can be used as a biomarker of psychopathology [for a review, see (19)].

Abnormal HRV has been proposed to be diagnostically helpful. There is some specificity in HRV profile in schizophrenia compared to bipolar disorder (26), PTSD, and MDD (27). Reduced HRV is related to social stress in those who are in their first-episode of schizophrenia (28), and is evident in unaffected relatives (29). However, HRV is not reliably abnormal in those who are at clinically high-risk of psychosis (i.e., those who are showing symptoms but have not had their first onset of psychosis) (30), reducing the viability of HRV being used as a marker of future psychiatric health.

Another issue to consider with HRV in schizophrenia is that low HRV can be a side effect of clozapine, a common comparator for all antipsychotic medication (31, 32). However, suppressed HRV was still evident in unmedicated patients and was exacerbated in medicated patients (33). Longitudinal studies reported improvements in HRV with medication (which may be related to symptoms) (34), and highlights the need for frequent HRV reporting before and during medication administration to examine the relationship with cognitive symptoms.

In autism, there is a similar reduction in HRV when compared to controls [meta-analyses (20, 21)]. The majority of the studies measuring HRV in autism focused on children and adolescents find relatively consistent effects. For the few reports in adults, similar reductions are reported (35), particularly when under social (36), or sensory stimulation (37), reflecting impaired emotion regulation (38), worse inhibitory control (39), and more symptoms (40). HRV has also been found to mediate task performance (speed at responding to a cue) and this relationship differs subtly between autism and ADHD suggesting some specificity with diagnosis (41). However, there is a report of no HRV reduction in adults with autism compared to controls during a public speaking task (36), while others suggest that HRV may be more suppressed during rest than during a cognitive task (42), or that situational changes in HRV may be more blunted in autism than in controls (43). Together, this suggests that the HRV profile in autism compared to schizophrenia may reveal unique underlying physiological differences.

The current study directly compared adults with schizophrenia, adults with autism, and neurotypical controls on three measures of HRV: standard deviation in R-R peaks (SDNN), low frequency power in the heartbeat, and high frequency power in the heartbeat. SDNN and low frequency power are commonly interpreted to reflect sympathetic functioning, whereas the high frequency power reflects both sympathetic and parasympathetic functioning (9).

The importance of ascertaining the HRV profile in adults with autism stems from the direct relationship with cognitive aging and increased cardiovascular risk. Autism is a lifelong condition, and so identifying markers of cognitive and physical health can assist with identifying who needs treatment and if the treatments are working. For the purposes of this study where we compare autism to schizophrenia, focusing on adults ensures that all participants have a diagnosis and are similar in age. Autism typically manifests during childhood, while schizophrenia typically manifests in late adolescence through early adulthood. Therefore, identifying unique characteristics for each diagnosis requires age-matching to avoid a major potential confound.

We recorded electrocardiography (ECG) as part of an electroencephalography (EEG) study. Due to the different symptom scales used in the diagnosis of schizophrenia and autism, we also included the UCLA Loneliness Scale (44) to provide a measure of social stress for all participants to assess any relationship between HRV and feelings of loneliness. We hypothesized that HRV would be reduced in schizophrenia and autism compared to controls with some subtle differences in HRV profile between schizophrenia and autism. We anticipated that the schizophrenia and autism groups would report feeling lonelier than controls, and that this difference may be related to the suppressed HRV.

## Methods and Materials

### Participants

Twelve adults with schizophrenia, 25 adults with autism, and 27 neurotypical controls participated. None of the participants reported any recent significant head injury or were pregnant. Participants gave their informed consent and were paid $50 for their time. This protocol was approved by the Institutional Review Board at Carnegie Mellon University.

The adults with schizophrenia all met the DSM-IV criteria for schizophrenia and had full IQ (Wechsler Abbreviated Scale of Intelligence; WASI) scores above 95 (45). Clinical diagnosis was confirmed with the Structured Clinical Interview for DSM-IV (SCIP-P) by expert clinicians at Western Psychiatric Hospital at the University of Pittsburgh Medical Center. None of the schizophrenia group had a known comorbid condition. All of the participants in the schizophrenia group had at least 5 years of illness and nine participants reported being on antipsychotic medication (four were on clozapine, two were on risperidone, one was on fluphenazine, one was on haloperidol, and one participant could not name their anti-psychotic medication).

The adults with autism had full IQ scores above 87 (WASI) (45) and all met DSM-IV or DSM-V criteria for autism. Clinical diagnosis was confirmed with the Autism Diagnostic Observation Schedule (ADOS) (46) by clinicians at the Center For Excellence in Autism Research at the University of Pittsburgh. For medications, none of the autism participants reported being on antipsychotics, eight were on anti-depressants, four were on medication for anxiety, five took medication to help improve attention and reduce irritability (ADHD-related symptoms), and two participants took medication for obsessive-compulsive behavior. Despite the medications used for individual symptoms, none of the autism group had any known comorbidities or secondary diagnoses. The autism and schizophrenia groups did not differ in their age or IQ.

The neurotypical controls were from the Pittsburgh area and were age- and gender-matched to the schizophrenia and autism participants. None of the controls were taking any medications that could impact neurological function at the time of the experiment.

Due to the difficulties in sociability being a defining characteristic of both schizophrenia and autism, all participants completed the Revised UCLA Loneliness Scale (R-UCLA) by Hughes at al. (44). Participants had to state how much they experience certain situations. There were 20 situations in total, such as “I lack companionship.” Responses were scored as following: “Never” (1), “Rarely” (2), “Sometimes” (3), “Often” (4). Questions 1, 4, 5, 6, 7, 19, 15, 16, 19, and 20 were reverse scored. Scores were then summed to create an index of loneliness where higher scores reflect greater feelings of loneliness. The questionnaire was conducted by pen and paper and filled out by the participant.

### ECG Data Acquisition

ECG was collected as part of an auditory EEG experiment designed to examine auditory mismatch negativity in individuals with autism compared to individuals with schizophrenia. A 64-channel BioSemi Active2 EEG system (Amsterdam, Netherlands) was used to collect the data. Two flat Ag-AgCl electrodes (4 mm diameter) were added to the mastoids, and an additional flat electrode was attached to the collarbone to detect heartbeat (ECG). Flat electrodes contained the same conductive EEG gel as the cap electrodes and were attached with adhesive stickers. All electrodes were recorded relative to the standard BioSemi CMS and DRL electrodes. Data were digitized at 512 Hz with a 24-bit A/D conversion.

### Procedure

ECG was collected while participants heard tones presented over earphones that differed in pitch [1,046.5 Hz (C6), 1,108.73 Hz (C#6), and 1,244.51 Hz (D#6) presented three or nine times before the pitch changed], and EEG responses were recorded. Participants were asked to ignore the tones and attend to a central fixation cross and press the spacebar on the keyboard when the cross changed color (16.6% of trials). This was to ensure that participants were awake and attending while the tones were presented. The task lasted around 20 min and ECG was recorded for the entire duration. Raw EEG data containing the ECG trace are available through OSF (osf.io/wsp4j).

### Data Analysis

**Figure 1:**
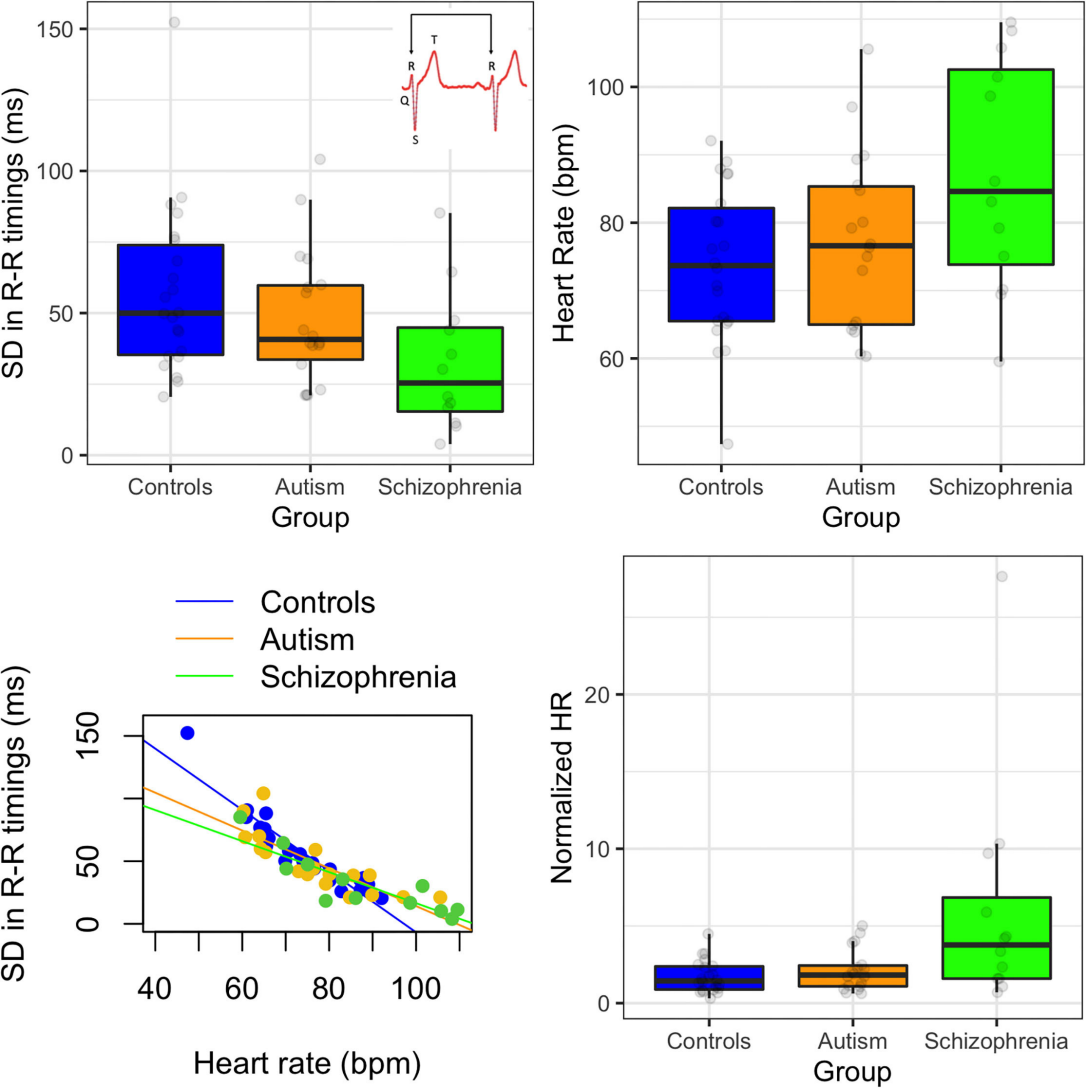
(Top left) Standard deviation in R-R peak timings in controls, autism, and schizophrenia; insert showing an example waveform with labeled peaks. (Top right) Heart rate in beats per minute (bpm) for each group. (Bottom left) relationship between heart rate and standard deviation in R-R timings. (Bottom right) normalized heart rate by variability for each group. Error bars show 95% confidence intervals.

ECG data were analyzed using MATLAB (MathWorks) and the EEGLAB toolbox extension (47). The following procedure was used to ensure high quality ECG data was collected: The ECG trace was re-referenced to the average mastoids and filtered between 0.1 and 100 Hz to remove slow wave drifts due to sweat potentials and excess muscle artifact. The fMRIB toolbox for EEGLAB was used to identify QRS peak onsets (see Figure 1 insert for ECG waveform and labeled peaks). Data 200 ms before the onset of the QRS complex to 500 ms after the onset of the QRS complex were extracted and baseline corrected. Any ECG traces that did not exhibit the expected ECG waveform were excluded from analysis: P amplitude was higher the Q, Q was lower than R, R was higher than S by at least 50 μV, S was lower than T, and the drift across the whole waveform was <200 μV. If more than 50% of ECG waveforms were rejected, then all the participant's ECG data were excluded from analysis. This resulted in seven adults with autism and five controls being removed from analysis. The final sample comprised 12 adults with schizophrenia (3 female, 9 male), 18 adults with autism (4 female, 14 male), and 22 neurotypical controls (6 female, 16 male). A summary of the demographic information and MATRICS scores for the final groups is shown in Table 1. A breakdown of the demographic and symptom information for the schizophrenia and autism groups are shown in Tables 2, 3, respectively. Groups did not differ significantly in their age, gender, IQ, or on any of the MATRICS subtests, when corrected for multiple comparisons. Analysis scripts and depictions of the ECG waveforms for each participant are included on GitHub (SarahMHaigh/HRV_AutismSchizophrenia).

**Table 1:** Summary demographic information and MATRICS scores for the schizophrenia, autism, and control groups.

	**Schizophrenia**	**Autism**	**Controls**	***P*-value**
Gender (M/F)	9/3	14/4	17/6	*p* = 0.935
Age (years)	34.17	29.78	29.18	*p* > 0.09
IQ	107.09	110.22		*p* = 0.525
BACS	27.70	24.26		*p* = 0.790
Processing speed	30.12	37.77		*p* = 0.535
Attention vigilance	36.26	38.23		*p* = 0.870
Working memory	42.74	45.25		*p* = 0.834
Verbal learning	21.05	41.66		*p* = 0.057
Visual learning	30.89	55.02		*p* = 0.052
Reasoning and problem solving	55.18	56.01		*p* = 0.951
Social cognition	44.58	38.64		*p* = 0.630

*Raw IQ and standardized MATRICS scores for the controls were not collected. All group comparisons were assessed using t-tests, except when comparing gender where a chi-square test was used*.

**Table 2:** Demographic information for the schizophrenia group.

**Participant**	**BPRS**	**IQ**	**Age**	**Gender**
1	37	109	30	F
2	38	101	28	F
3	52	114	37	M
4	38	99	25	M
5	48	104	30	M
6	39	100	48	M
7	33	121	37	M
8	39	107	46	M
9	47	99	32	M
10	60	95	32	F
11			24	M
12	30	129	41	M

*For one individual with schizophrenia, the Brief Psychiatric Rating Scale Score (BPRS), and IQ information was missing*.

**Table 3:** Demographic information for the autism group.

**Participant**	**ADOS communication**	**ADOS stereotypical**	**IQ**	**Age**	**Gender**
1	3	7	92	28	F
2	8	9	111	21	M
3	4	10	87	44	M
4	4	7	104	29	M
5	2	7	123	38	F
6	2	5	128	32	M
7	4	4	128	41	M
8	3	8	123	29	M
9	2	9	114	39	F
10	5	9	115	19	M
11	2	5	107	22	M
12	3	5	89	26	M
13	3	4	88	31	M
14	2	6	120	36	F
15	5	5	100	41	M
16	2	5	99	22	M
17	5	9	127	19	M
18	4	4	129	19	M

The ECG data were reanalyzed to calculate the HRV measures. The ECG trace was re-referenced to the average mastoids and filtered between 0.1 and 100 Hz to remove slow wave drifts due to sweat potentials and excess muscle artifact. The fMRIB toolbox was used to identify the beginning of QRS complex. The timing of the largest positive peak after the onset of the QRS complex was identified (the R-peak). The standard deviation in the difference in R-peak timings was then calculated (SDNN). We chose to focus on the SDNN measure [as opposed to the root mean squared difference; RMSSD; or the variability across 5-min time intervals, see (48) for a review] as SDNN equally reflects variability across the cardiac cycle (as opposed to RMSSD which is biased toward the high frequency vagal cycle) (49). To verify that our rejection criteria described above did not skew the results, the SDNN were reanalyzed to include the ECG responses that did not conform to the expected waveform. The results were weaker but showed the same effects as described below.

As SDNN is known to negatively correlate with heart rate (HR; those with higher HR exhibit reduce SDNN) (50), we also analyzed group differences in the number of beats per minute (bpm) by calculate the number of beats over the duration of the recording. We then calculated the normalized HR by dividing the bpm by SDNN for each participant [as recommended by (50)] and assessed if the group differences still held.

To assess high and low frequency power in the ECG trace, the raw ECG data were filtered at 0.04–100 Hz and run through a Fast Fourier Transform (FFT). We analyzed the raw data for the frequency analyses (as opposed to the corrected data from the SDNN analysis) to ensure that chopping up the missing data did not introduce artifacts in the FFT results. The low frequency power measure was calculated between 0.04 and 0.15 Hz and the measure of high frequency power was calculated between 0.15 and 0.4 Hz. The cutoffs are in line with previous reports of high and low frequency power in the ECG trace in autism and schizophrenia populations (35, 49).

The differences in high frequency ECG power, low frequency ECG power, SDNN, and finally in loneliness scores, were analyzed separately using independent samples ANOVA so that the schizophrenia, autism, and control groups could be directly compared. Pairwise comparisons were used to evaluate group differences and were Bonferroni corrected for multiple comparisons. Exploratory Spearman's correlations were run to compare HRV and symptom measures. As both autism and schizophrenia groups completed the MATRICS Consensus Cognitive Battery, the age and gender corrected percentiles of the composite scores in the main cognitive domains were correlated with HRV [the Brief Assessment of Cognition in Schizophrenia (BACS), processing speed, attention vigilance, working memory, verbal learning, visual learning, reasoning and problem solving, and social cognition]. Two individuals with autism and one individual with schizophrenia did not complete the MATRICS battery and two additional individuals with autism only completed the BACS, processing speed, and attention vigilance sections of the MATRICS battery.

## Results

First, we assessed differences in the standard deviation in R-R peak timings (SDNN). There were significant differences between groups [*F*_(2, 49)_ = 3.41, *p* = 0.041] due to the schizophrenia group producing smaller SDNN than controls (*p* = 0.041). The autism group produced nominally smaller SDNN than controls but this effect was not significant (*p* = 0.264). Heart rate (HR) was also significantly different between groups [*F*_(2, 49)_ = 3.94, *p* = 0.026], due to nominally elevated HR in the schizophrenia group compared to controls, but this was not significant in *post-hoc* comparisons (*p*s > 0.05). HR was negatively correlated with SDNN [*r*_*s*__(50)_ = −0.93, *p* < 0.001], and this was consistent for all three groups (*r*_*s*_ < −0.90). When HR was normalized by SDNN, then the differences between the groups increased [*F*_(2, 49)_ = 5.87, *p* = 0.005]. There was one outlier in the schizophrenia group (see Figure 1) but when this individual was removed from analysis, the group effects remained the same.

**Figure 2:**
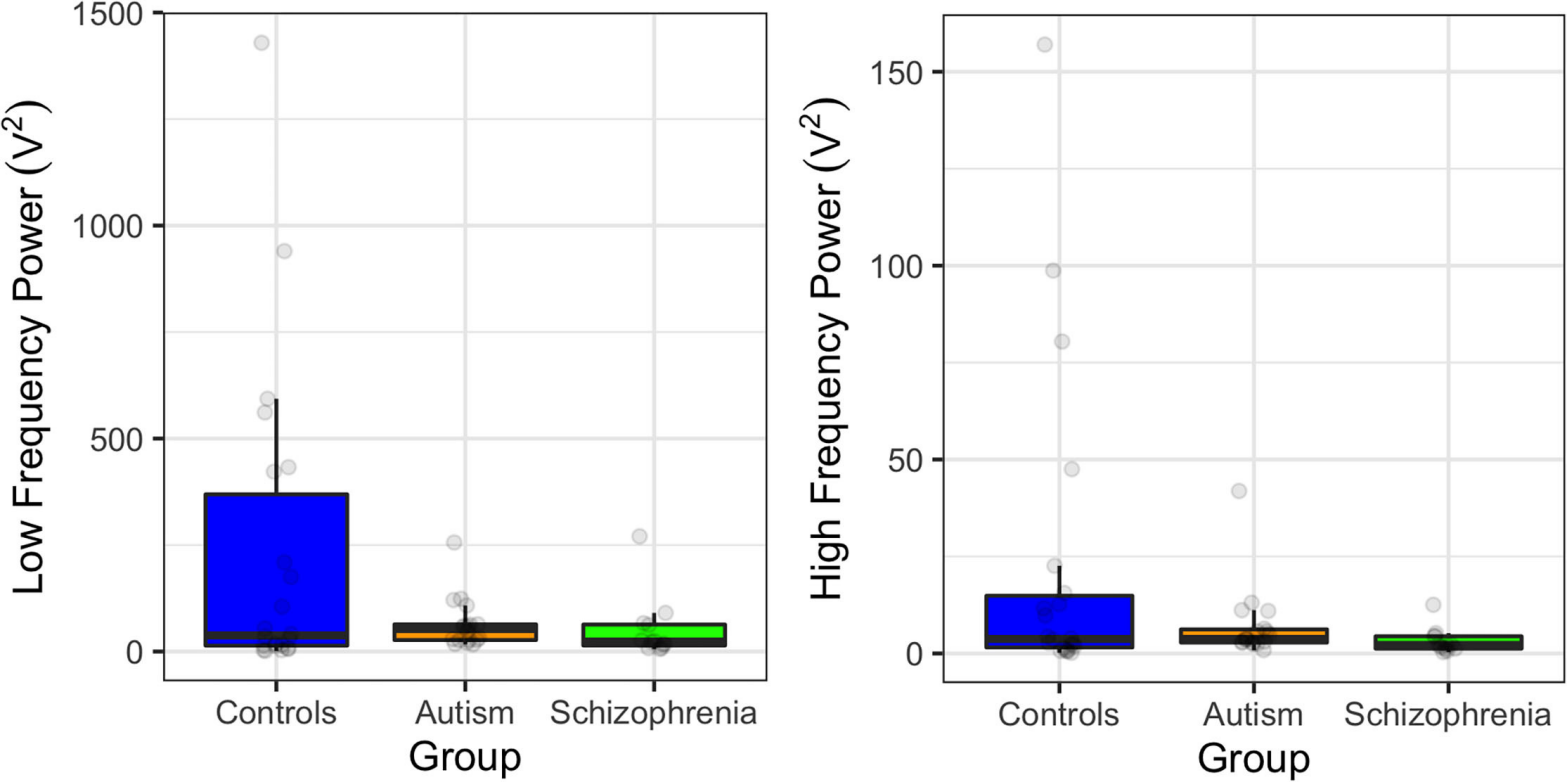
Power at low frequencies (0.04–0.15 Hz; left) and at high frequencies (0.15–0.4 Hz; right) in controls, individuals with autism, and individuals with schizophrenia. Error bars show 95% confidence intervals.

When assessing group differences in power, there were marginally significant differences in low frequency power [*F*_(2, 49)_ = 3.14, *p* = 0.052] due to the controls exhibiting greater low frequency power compared to schizophrenia and autism, but this did not survive Bonferroni correction (*p* = 0.110; *p* = 0.140, respectively). However, there were no group differences in high power [*F*_(2, 49)_ = 2.42, *p* = 0.099; Figure 2]. Owing to the previous associations between HRV and antipsychotic medication, the three unmedicated participants with schizophrenia were removed and the analyses were rerun. Removing the unmedicated participants weakened the difference between schizophrenia and control participants, suggesting that the medications were not primarily driving these effects [SDNN: *F*_(2, 49)_ = 2.04, *p* = 0.141; low frequency power: *F*_(2, 49)_ = 3.14, *p* = 0.053; *F*_(2, 49)_ = 2.19, *p* = 0.123].

### Relationships Between HRV and Symptom Severity

When assessing group differences in feelings of loneliness, there was a marginally significant difference between groups [*F*_(2, 49)_ = 3.05, *p* = 0.057], where adults with autism reported feeling more lonely than controls. Loneliness scores were similar, on average, between schizophrenia and controls. Interestingly, loneliness scores did not significantly correlate with any of the HRV measures for any of the groups individually or across all individuals (0.18 > *r* < −0.16).

Owing to the stronger group differences in SDNN compared to the marginal differences in low frequency power, only correlations between SDNN and symptom and cognitive scores were assessed. This served to reduce the number of comparisons made. There were no significant correlations between symptom measures (ADOS stereotypical behavior and communication scores) and SDNN in autism, nor between scores on the Brief Psychiatric Rating Scale and SDNN in schizophrenia. There were also no significant correlations between IQ score and SDNN for the autism and schizophrenia groups independently or when pooled together. IQ was not collected for controls and so could not be assessed.

**Figure 3:**
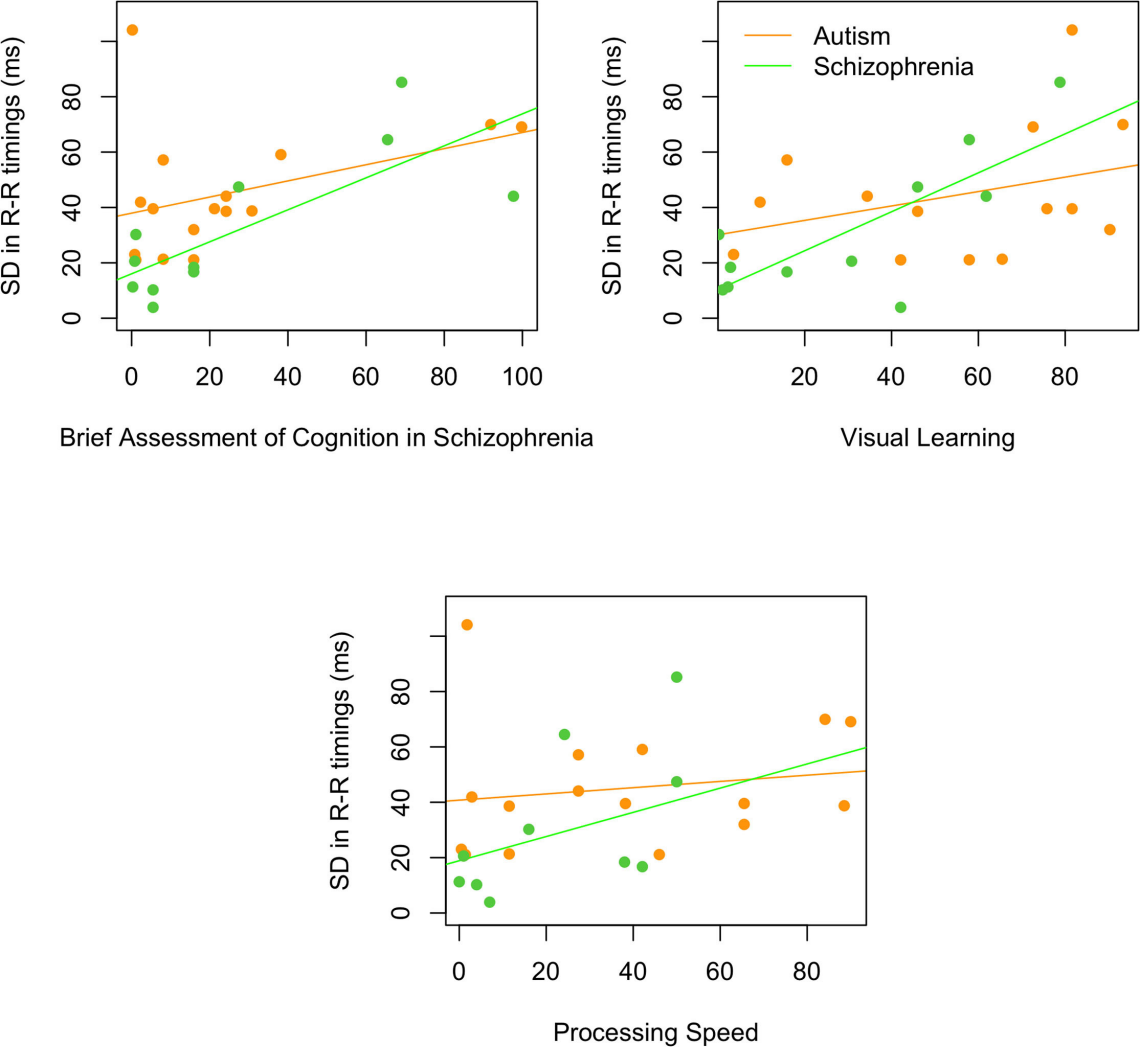
Relationships between SDNN and the Brief Assessment of Cognition in Schizophrenia, visual learning, and processing speed. All correlations were significant in the schizophrenia (green) and not the autism (orange) group.

For the composite MATRICS scores, better performance on the Brief Assessment of Cognition in Schizophrenia was related to higher SDNN [*r*_*s*__(25)_ = 0.54, *p* = 0.004] that was primarily driven by the individuals with schizophrenia [*r*_*s*__(9)_ = 0.66, *p* = 0.028; autism: *r*_*s*__(14)_ = 0.34, *p* = 0.197]. Similarly, better performance on visual learning was associated with higher SDNN [*r*_*s*__(23)_ = 0.59, *p* = 0.002], due to a stronger correlation in schizophrenia than in autism [schizophrenia: *r*_*s*__(9)_ = 0.64, *p* = 0.035; autism: *r*_*s*__(12)_ = 0.28, *p* = 0.337]. However, faster processing speed was associated with lower SDNN [*r*_*s*__(23)_ = 0.45, *p* = 0.018; schizophrenia: *r*_*s*__(9)_ = 0.63, *p* = 0.038; autism: *r*_*s*__(12)_ = 0.29, *p* = 0.278]. Figure 3 shows the correlations for both autism and schizophrenia.

## Discussion

Abnormal heart rate variability (HRV) has been associated with several psychiatric conditions including schizophrenia and autism. Directly comparing the HRV profile across diagnoses can identify properties that are unique to one diagnosis and can serve as a future biomarker. The current study found that HRV markers, specifically standard deviation in the timing of the R-R peaks (SDNN) and power in the low frequency band, were most suppressed in schizophrenia, and less so in autism, compared to controls. This suggests that HRV is most impacted in schizophrenia and could represent unique pathology. In addition, these effects did not seem to be driven by the antipsychotic medication—if they were, then removing the three unmedicated individuals with schizophrenia should have strengthened (and not weakened) the group differences.

Reduced SDNN and low frequency power measures of HRV are commonly believed to be due to suppressed autonomic functioning, which often occurs in response to chronic stress (51, 52). The higher heart rate and normalized HR in schizophrenia is also consistent with an increased stress response. Stress has been highlighted as a key component for causing or exacerbating symptoms in psychiatric individuals and increases the likelihood of diagnosis. For example, episodes of acute stress such as a death in the family or bullying during development are significant predictors of psychopathology onset and reoccurrence [for a review, see (53)]. Stress markers predict quality of life and are mediated by coping strategies, suggesting behavioral intervention would improve some of the effects of stress [schizophrenia (54); autism (55)]. Stress during critical periods of development can exacerbate physiological and psychological symptoms and increase stress sensitivity through adulthood (56). Therefore, tracking markers of stress may be helpful for determining neurological and physiological health transdiagnostically throughout the lifespan. HRV is a simple and cost-effective method of doing so.

It is surprising then, that there was a lack of significant (or consistent) correlations between HRV and feelings of loneliness, suggesting that there is not a simple direct relationship between perceived social stress and autonomic functioning. Furthermore, despite deficits in sociability being a defining characteristic of both autism and schizophrenia, only the autism group on average reported feeling lonely. The schizophrenia group on average reported similar feelings of loneliness as controls. This surprising result highlights a discrepancy between the characteristics that define a diagnosis and what the individual perceives.

On the other hand, correlations between HRV (specifically SDNN) and measures of cognition from the MATRICS battery show that worse cognitive ability was associated with lower SDNN, and that this was particularly the case for the schizophrenia group. It is possible that the reduced HRV is reflecting neurocognitive factors and that generalized improvements in cognition with training or treatment may also increase HRV.

Interestingly, there were no obvious relationships between HRV and symptomology in either the autism or schizophrenia groups. This could mean that the symptom measures capture too complex behavioral patterns to be able to accurately reflect underlying physiology. One point to note, is that while symptoms such as deficits in social interaction and communication are known to be present in both autism and schizophrenia diagnoses (identified using the ADOS and SCID), we did not examine these deficits using the same measure. It may be of interest to examine the prevalence of autism and schizophrenia-related symptoms across all participants, including the controls, to further examine the relationship between symptoms and physiology.

Interestingly, we did not see evidence of abnormal high frequency power in schizophrenia or in autism. High frequency power is commonly linked to both sympathetic and parasympathetic functioning, while low frequencies and SDNN are linked to sympathetic functioning alone (9), although there is some suggestion that low frequency power is more dominated by sympathetic functioning too (48). Suppressed high frequency power (interpreted as suppressed parasympathetic functioning) has been found in autism previously (35), and deficits in stress response recovery in schizophrenia have also implicated worse parasympathetic functioning (19). There is some debate as to how helpful and accurate the relationships between HRV measures and specific autonomic functioning are (57). It is possible that one branch of the autonomic system (either the sympathetic or parasympathetic nervous system) is more associated with psychiatric functioning that the other, but this division in not clear in the HRV measures.

### Limitations

There are several limitations to this study to address. The first is that ECG was measured during an auditory EEG experiment where participants were listening to tones while completing a simple visual attention task. While this study was not cognitively demanding, it was also not at rest either, making it difficult to generalize these findings across other resting or task-related studies. It is important to note that the HR reported in Figure 1 shows that HR across all participants was between 47 and 110 bpm, which is consistent with resting heart rate (58) and so is more likely to be comparable to other resting HRV studies. Second, because the ECG was recorded during an EEG study, only one electrode was used to record the ECG data. Typically, at least three active leads are used to record ECG and so the single electrode may have been less stable. To ensure that noisy data were not included in the analyses, we used additional exclusion criteria where ECG waveforms that did not conform to the expected QRS peaks were rejected and participants' data were excluded from analyses if more than 50% of data did not meet these criteria. Third, the strict exclusion criteria resulted in data from seven adults with autism being rejected from analyses. While visual inspection of the data suggested that the channel itself was noisy for these individuals (data are available through OSF; osf.io/wsp4j), it is possible that the ECG waveforms themselves were abnormal in these individuals (perhaps due to poor electrodermal connections or excess motion) which could be an interesting finding by itself. Repeating this study with a multi-lead ECG system would verify if this were the case. Fourth, we did not collect IQ scores from our control participants. While there are no significant correlations between IQ and HRV in the autism and schizophrenia groups, there may be small effects of IQ that contribute to the group differences. Fifth, the schizophrenia group in particular was small. Despite the reduced statistical power from having small sample sizes, we were still able to detect HRV reductions in schizophrenia which is consistent with the literature. However, these findings should be approached with caution until replicated.

In summary, HRV offers a simple measure of autonomic functioning that can help distinguish between psychiatric and neurotypical individuals. The finding that reduced HRV was only significantly lower in schizophrenia suggests that there are subtle differences in HRV profiles between different conditions that may be linked to neurological and physiological symptomology and long-term health.

## Data Availability Statement

The datasets presented in this study can be found in online repositories. The names of the repository/repositories and accession number(s) can be found below: osf.io/wsp4j/.

## Ethics Statement

The studies involving human participants were reviewed and approved by Institutional Review Board, Carnegie Mellon University. The patients/participants provided their written informed consent to participate in this study.

## Author Contributions

SH designed the study, analyzed the data, and wrote the manuscript. TW analyzed the data and edited the manuscript. PB collected the data and edited the manuscript. All authors contributed to the article and approved the submitted version.

## Funding

This project was supported by a NARSAD Young Investigator Grant from the Brain and Behavior Research Foundation (26282) to SH, an R15 AREA award from the National Institute of Mental Health (122935) to SH, an NSF EPSCoR grant (1632849) on which SH was a Co-Investigator.

## Conflict of Interest

The authors declare that the research was conducted in the absence of any commercial or financial relationships that could be construed as a potential conflict of interest.

## Publisher's Note

All claims expressed in this article are solely those of the authors and do not necessarily represent those of their affiliated organizations, or those of the publisher, the editors and the reviewers. Any product that may be evaluated in this article, or claim that may be made by its manufacturer, is not guaranteed or endorsed by the publisher.
